# A Single Codon Optimization Enhances Recombinant Human TNF-*α* Vaccine Expression in* Escherichia coli*

**DOI:** 10.1155/2018/3025169

**Published:** 2018-04-15

**Authors:** Chu Chu, Wangqian Zhang, Jialin Li, Yi Wan, Zenglu Wang, Ruyi Duan, Pei Yu, Ning Zhao, Kuo Zhang, Shuning Wang, Qiang Hao, Weina Li, Cun Zhang, Wei Zhang, Yingqi Zhang, Meng Li, Xiaochang Xue

**Affiliations:** ^1^State Key Laboratory of Cancer Biology, Department of Biopharmaceutics, School of Pharmacy, Fourth Military Medical University, Xi'an 710032, China; ^2^Shaanxi Institute of Microbiology, Xi'an 710043, China; ^3^Department of Pharmacogenomics, School of Pharmacy, Fourth Military Medical University, Xi'an 710032, China

## Abstract

As a proinflammatory cytokine, tumor necrosis factor-alpha (TNF-*α*) plays a pivotal role in various autoimmune diseases such as rheumatoid arthritis (RA). Thus, TNF-*α* has been defined as a therapeutic target for RA. Although some TNF-*α* antagonists including neutralizing monoclonal antibodies and soluble receptors have been approved to be successful in attenuating symptoms in patients suffering from RA, the long-term use of these passive immunization reagents could cause some problems like a variable degree of immunogenicity. In the present study, in order to wake up active immune responses of RA patients, we developed a recombinant TNF-*α* therapeutic vaccine (named mrTNF-PADRE) by coupling a 12-amino acid universal Pan HLA-DR Epitope (PADRE) to the protein. Codon optimization was performed to improve the secondary structure of mrTNF-PADRE mRNA to ensure its heterologous expression. As a result, a single codon synonymous mutation greatly elevated recombinant protein expression (about 30% of the total bacteria proteins) in* E. coli* as compared with the undetectable expression of the unoptimized gene. Although expressed as insoluble inclusion bodies (IBs), the vaccine can be effectively prepared with a purity of over 95% by IBs washing and one-step gel-infiltration chromatography. By this strategy, a stable yield of 5.2 mg purified mrTNF-PADRE per gram of cell paste could be obtained.

## 1. Introduction

It is well known that TNF-*α* belongs to a structurally homologous family of cytokines which can fold to be a homotrimer assuming a “jelly roll” conformation and plays a critical role in immune homeostasis [1, 2]. By binding with two distinct membrane receptors, TNFR-1 and TNFR-2, TNF-*α* can potently regulate inflammatory responses-mediated gene expression and cellular apoptosis and necrosis. However, aberrant TNF-*α* overexpression can induce chronic inflammation and subsequent tissue destruction and thus play important roles in various diseases including cachexia, Crohn's disease (CD), and rheumatoid arthritis (RA) [3, 4].

By neutralizing the endogenous TNF-*α*, some anti-TNF-*α* monoclonal antibodies (mAb, such as etanercept and infliximab) and soluble TNF-*α* receptor have been successfully used to attenuate pain and symptoms in patients suffering from CD and RA [5–7]. However, expensive price, repeated administration, and usually only aiming at one epitope of TNF-*α* greatly limited their practical applications [7, 8]. Many RA patients failed to respond to these antagonists because of genetic polymorphism [9, 10]. In addition, some studies uncovered that the known immunosuppressive effects of the anti-TNF-*α* drugs provide a predisposition of patients with RA and CD to lymphoma, although a causal relationship between them still cannot be established [11–13].

The strategy to develop protein autovaccines by coupling an exogenous T helper cell epitope has emerged recently and attracted much attention [14]. This active vaccination strategy overcomes the shortcomings of passive neutralizing mAb administration and takes advantage of the overactive immune systems of RA patients and educates it to produce polyclonal neutralizing antibodies before reaching a new immune system homeostasis. In addition, repeat agents administration can be avoided and the induced polyclonal antibodies can broaden the anti-TNF-*α* spectrum.

Here, we constructed a recombinant TNF-*α* vaccine by fusion expressed TNF-*α* with the T helper cell epitope PADRE. The designated mrTNF-PADRE vaccine was expressed in* Escherichia coli (E. coli)* at high level after mRNA secondary structure optimization by a single codon synonymous mutation. The recombinant vaccine can be conveniently purified by IBs washing and one-step gel filtration chromatography. The final yields were about 5.2 mg purified mrTNF-PADRE per gram cell paste.

## 2. Materials and Methods

### 2.1. Reagents

Restriction enzymes, T4 DNA ligase, and Phusion High-Fidelity DNA Polymerase were purchased from New England Biolabs (Ipswich, MA). Oligonucleotides were synthesized by Beijing AuGCT Biological Technology Co., Ltd (Beijing, China). The vector pET-22b and* E. coli *strain BL21 (DE3) were purchased from Novagen (San Diego, CA). Isopropyl *β*-D-1-thiogalactopyranoside (IPTG) was from Sigma-Aldrich (St. Louis, MO). Sephacryl S-300 was from GE Healthcare Life Sciences (Uppsala, Sweden). Mouse anti-human TNF-*α* monoclonal antibody and HRP-conjugated rabbit anti-mouse IgG were from Abcam (UK, Cambridge). Standard recombinant hTNF-*α* was purchased from National Institutes for Food and Drug Control (Beijing, China). The fermentor was from B. Braun (Germany). All other reagents were of analytical grade.

### 2.2. Construction of pET-22b-(m)rTNF-PADRE Plasmids

To construct human TNF-*α* autovaccine, the antigenic determinants of TNF-*α* were firstly predicted by Antigenic software based on the method of Kolaskar and Tongaonkar [15] and the autovaccine rTNF-PADRE* (recombinant TNF-PADRE)* was constructed according to our previous strategy [3], in which a T helper epitope PADRE was cloned into the wild-type TNF-*α* gene and replaced the TNF-*α*_127–141_ fragment. In order to splice the recombinant gene conveniently, two synonymous mutations were introduced into wild-type TNF-*α* gene and the sequence CAGCTG that encodes amino acid residues 125 and 126 (Gln-Leu) was mutated to CAATTG, the restriction enzyme Mun I recognition sequence. Then, the DNA coding for PADRE (AKFVAAWTLKAA) and the C-terminus fragment of TNF-*α* (TNF-*α*_142–157_) were obtained by oligonucleotide synthesis according to the sequences PTF and PTR listed in Table 1. The sequences were designed to include Mun I (5′ end) and Sal I (3′ end) restriction sites (underlined). The gene of TNF-*α*_1–126_ was amplified from TNF-*α* gene with the primers P1 and P2 (Table 1). Nde I and Mun I restriction sites are underlined. The PADRE oligonucleotides (annealed) and PCR products were then cloned into pET-22b vector digested with Nde I and Sal I. The construct of resulting plasmid pET-22b-rTNF-PADRE was confirmed by DNA sequencing.

For codon optimization, the rTNF-PADRE gene was obtained from pET-22b-rTNF-PADRE plasmid with the primers P3 and P4 (Table 1). Nde I and Sal I restriction sites are underlined and the optimized nucleotides were double underlined. Finally, the constructed plasmid pET-22b-mrTNF-PADRE* (mutant recombinant TNF-PADRE)* was confirmed by DNA sequencing.

### 2.3. Expression of Recombinant mrTNF-PADRE Vaccine in* E. coli*

To express the recombinant vaccine TNF-PADRE in* E. coli*, pET-22b-rTNF-PADRE and pET-22b-mrTNF-PADRE vectors were transformed into BL21 (DE3) competent cells, respectively, and positive clones were determined by DNA sequencing. A single colony was then inoculated into 5 mL LB medium containing 100 *μ*g/ml of ampicillin and cultured with 200 rpm shaking at 37°C overnight. The bacteria cultures (3 ml) were then transferred to 300 ml fresh LB medium and grown to an OD_600_ of 0.5. For protein expression, 1 mM IPTG (final concentration) was added and samples (1 ml) were collected 4 h later and analyzed by SDS-PAGE. For protein purification, bacteria were cultured in large scale with a 5 L fermentor as previously described [16]. The culture was harvested by centrifugation at 12,000 rpm for 30 min at 4°C and cell pellets were stored at −20°C for later use.

### 2.4. Western Blot Analysis

Western blot was performed as previously described [16]. In brief, proteins were transferred to polyvinylidene fluoride membranes (0.22 *μ*m; Invitrogen, USA) and Western blot analyses were carried out using an hTNF-*α* monoclonal antibody, followed by an HRP-labeled IgG. The target proteins were finally visualized with an enhanced chemiluminescence (ECL, Thermo Fisher Scientific, USA).

### 2.5. Purification of mrTNF-PADRE

mrTNF-PADRE was purified as previously described but optimized [3]. In brief, 100 g (wet weight) of cell pellet was suspended with 500 ml of lysis buffer (20 mM Tris-HCl, pH 8.5, 150 mM NaCl, 1 mM EDTA) and thoroughly disrupted with an ultrasonic crusher (300 W, 25 min). After being centrifuged at 12,000 rpm at 4°C for 25 min, the inclusion bodies (IBs) were washed twice with the washing buffer (1000 ml of 4 M urea, 1% Triton X-100, 5 mM EDTA, 5 mM *β*-mercaptoethanol, 50 mM Tris-HCl pH 8.5) at room temperature for 30 min. The final pellet of IBs was solubilized by homogenizing in 20 mM *β*-mercaptoethanol and 7 M guanidine HCl. The supernatant was then loaded onto a chromatography column (2.4 × 100 cm) packed with 500 ml of depyrogenated Sephacryl S-300 resin and equilibrated with 50 mM Tris-HCl, pH 8.5, 8 M urea, and 150 mM NaCl. To purify mrTNF-PADRE, the column was eluted with the equilibration buffer at a flow rate of 1.0 ml/min (ÄKTA purifier, GE Healthcare). The purified mrTNF-PADRE fraction was then refolded by dialyzing against the refolding buffers (10 mM Tris-HCl, pH 8.5 supplement with 1/1000 (v/v) *β*-mercaptoethanol, 1/10^5^ (v/v) 0.5 M CuSO_4_) at 20°C for 8 h and then against PBS at 20°C for 8 h twice. After removing the aggregated particles by centrifugation, the final products were lyophilized and stored at −70°C.

### 2.6. Characterization of mrTNF-PADRE

Size exclusion chromatography- (SEC-) HPLC analyses were performed on a Waters HPLC apparatus. The sample (5 *μ*g) in PBS was injected onto a 7.5 mm × 300 mm G2000SW column (TOSOH) at a flow rate of 0.5 ml/min. Peaks were detected by monitoring at a wavelength of 275 nm. The purity of mrTNF-PADRE was calculated as a percentage of the total peak area. N-terminus amino acid sequencing was finished by Shanghai Shenggong Biological Engineering Co., Ltd. (Shanghai, China).

### 2.7. Immunization of Animals with mrTNF-PADRE

The recombinant vaccine was used to immunize mice with the protocol as previously described [17]. In brief, 80 *μ*g of mrTNF-PADRE formulated in saline was used to immunize C57BL/6 mice (*n* = 6) subcutaneously (s.c.) without adjuvant, and saline was used as the negative control. Sera were collected 10 days after the last immunization and antibody titers were analyzed by enzyme-linked immunosorbent assay (ELISA).

### 2.8. Neutralization Assay

For the* in vitro* neutralization assay, we detected the ability of antisera to inhibit TNF-*α*-stimulated fibroblast-like synoviocytes (FLS) proliferation. FLS cells were separated from the synovial tissues of RA patients and cultured as previously described [4]. FLS cells were stimulated with TNF-*α* (100 ng/mL), either alone or in the presence of the antisera, and then cultured at 37°C with 5% CO_2_ in humid air. Normal mouse serum was used as a negative control. Cell proliferation was measured with a BrdU assay kit (Roche, Basel, Switzerland) according to the manufacturer's protocol. The absorbance was measured at 450 nm using a multiwell microplate reader.

## 3. Results and Discussion

### 3.1. Optimization and Construction of (m)rTNF-PADRE Gene

**Figure 1:**
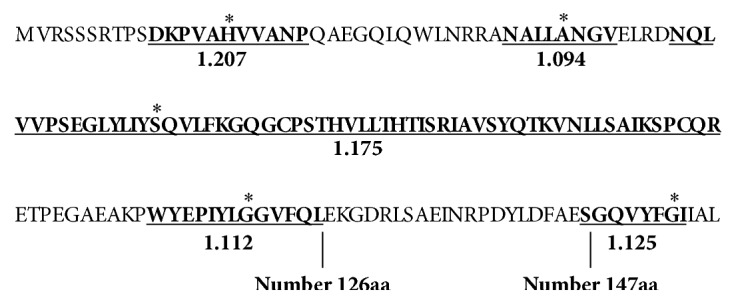
Prediction of antigenic determinants in human TNF-*α* by antigenic software. All the theoretical antigenic determinants were underlined and the scores were labeled accordingly. *∗* shows the maximum score positions in each of the antigenic determinants. PADRE helper T cell epitope was cloned between numbers 126 and 147 amino acid residues.

**Figure 2:**
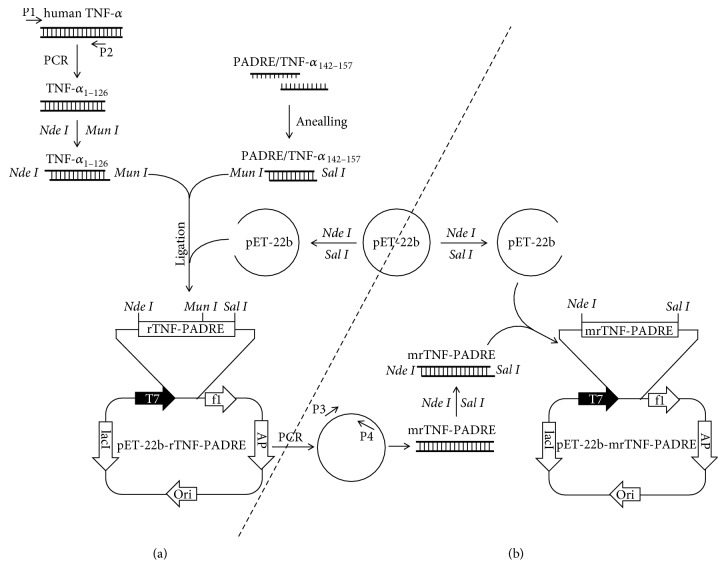
The sketch maps of rTNF-PADRE and mrTNF-PADRE construction. The sequences of P1–P4 primers are explained in Materials and Methods.

To obtain recombinant human TNF-*α* vaccine, we firstly predicted the antigenic determinants of TNF-*α* by antigenic software based on the method of Kolaskar and Tongaonkar [15]. As shown in Figure 1, five antigenic determinants (numbers 10–21, 34–41, 46–103, 114–126, and 147–154 fragments, resp.) were found in TNF-*α*. Thus, the PADRE T helper epitope was designed to be cloned between residues 126 and 147 of TNF-*α* without destroying its antigenic determinants.  Figure 2 shows the sketch map of the rTNF-PADRE gene construction in which the sequence that encodes the amino acid residues 127–141 of wild type TNF-*α* was replaced by PADRE. However, this recombinant vaccine failed to be expressed in* E. coli* (Figure 4(a)).

**Figure 3:**
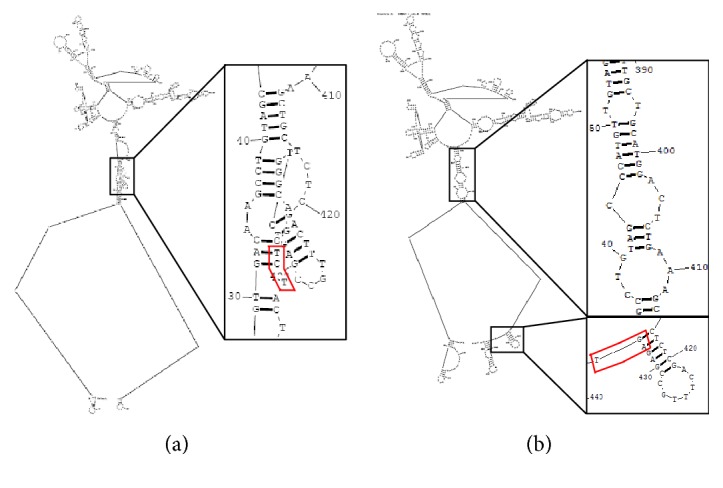
The secondary mRNA structure of vaccines rTNF-PADRE and mrTNF-PADRE. (a) The mRNA structure of rTNF-PADRE gene. (b) The mRNA structure of codon optimized mrTNF-PADRE gene in which a synonymous mutation was introduced. Black box showed the partially magnified structures and red box showed the synonymous mutation (TCT→AGT which encodes number 147 amino acid residue of wild-type human TNF-*α*).

After analyzing the rTNF-PADRE gene with software, we found that the secondary structure of its mRNA is folded in a very complicated manner which may suppress translation complex entry and movement through the mRNA chain (Figure 3(a)). To attain a high-level expression of the vaccine in* E. coli*, codon optimization was performed by PCR and a synonymous mutation (TCT was mutated to AGT which encodes number 147 amino acid residue of TNF-*α*) was introduced into the reverse primer P4 as shown in Figure 2. This single codon mutation greatly optimized the secondary mRNA structure of mrTNF-PADRE which is completely composed of stretched hairpin structures and is easy to be loosen and translated (Figure 3(b)). The optimized gene was then cloned into pET-22b vector and the correct plasmid was designated as pET-22b-mrTNF-PADRE.

### 3.2. Expression of mrTNF-PADRE

**Figure 4:**
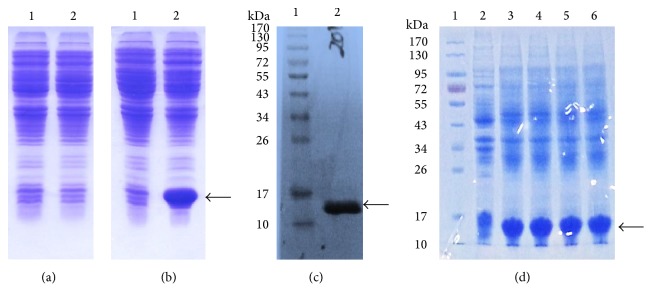
Expression and identification of TNF-PADRE. A and B plasmids pET-22b-rTNF-PADRE (a) and pET-22b-mrTNF-PADRE (b) were transformed into* E. coli *BL21 (DE3) strain and recombinant vaccine expression was induced by IPTG and detected by SDS-PAGE. Lane 1, total bacterial proteins before IPTG induction; lane 2, total bacterial proteins after IPTG induction. (c) Identification of expressed mrTNF-PADRE by Western blot. Lane 1, molecular weight standards (kDa); lane 2, total bacterial proteins after IPTG induction. (d) BL21 (DE3)/pET-22b-mrTNF-PADRE bacteria were cultured in a 5-L fermentor and mrTNF-PADRE expression was detected by SDS-PADRE. Lane 1, molecular weight standards (kDa); lane 2, total bacterial proteins without induction; lane 3–6, total bacterial proteins after 1–4 h induction. Arrowheads indicate the target protein.

For mrTNF-PADRE expression, the correct plasmid pET-22b-mrTNF-PADRE was transformed into BL21 (DE3) strain. The recombinant colonies BL21 (DE3)/pET-22b-mrTNF-PADRE were cultured in 5 ml LB medium and mrTNF-PADRE expression was induced by IPTG. As shown in Figure 4(b), a new band about 15.0 kDa was produced in the insoluble bacterial proteins with IPTG induction when compared with noninduced negative controls. The samples were further identified with Western blot and the new band was specifically recognized by anti-human TNF-*α* monoclonal antibody (Figure 4(c)). This indicates that mrTNF-PADRE was successfully expressed. Then, the bacteria were cultured in a fermentor and mrTNF-PADRE expression was detected by SDS-PAGE and expression level was analyzed with the BANDSCAN software (Figure 4(d)). Results showed that the new band accounts for about 30% of the whole cell lysate (data not shown).

### 3.3. Purification of mrTNF-PADRE

**Figure 5:**
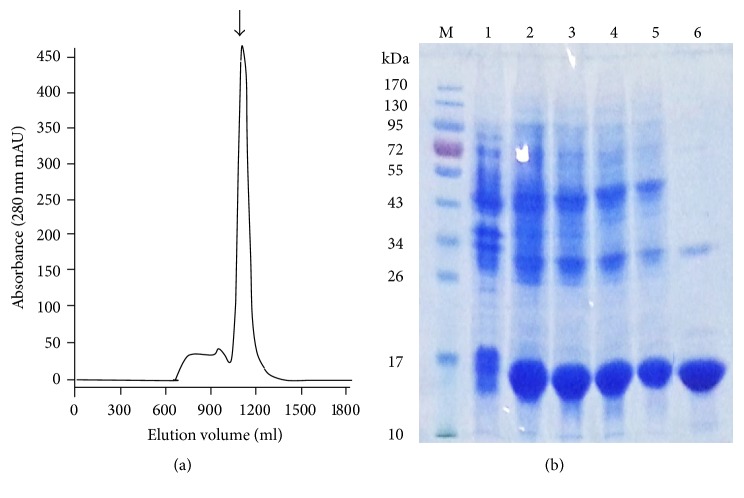
Purification of mrTNF-PADRE by gel filtration chromatography. (a) The elution profiles of Sephacryl S-300 chromatography for mrTNF-PADRE purification. Arrowheads indicate the mrTNF-PADRE fraction. (b) Analysis of mrTNF-PADRE purification by SDS-PAGE. Lane M, molecular weight standards (kDa); lane 1, total bacterial lysate without IPTG induction; lane 2, lysate of inclusion bodies with IPTG induction before washing; lanes 3 and 4, lysate of inclusion bodies after washing twice with the washing buffer; lane 5, supernatant of 7 M guanidine solubilized inclusion bodies; lane 6, purified mrTNF-PADRE after gel filtration chromatography.

One hundred grams of cell pellets was suspended in lysis buffer and disrupted with sonication and IBs were collected. After washing twice with the washing buffer, the IBs were then solubilized with 7 M guanidine HCl and 20 mM *β*-mercaptoethanol at 4°C overnight. The solubilized mrTNF-PADRE was purified by gel filtration chromatography with Sephacryl S-300 resin (Figure 5(a)). The purified mrTNF-PADRE fraction (about 0.4 mg/ml) was refolded by dialyzing against refolding buffers and then against PBS. The samples of each step were collected and analyzed by SDS-PAGE (Figure 5(b)). The final yields were 5.2 mg mrTNF-PADRE per gram cell paste (Table 2).

### 3.4. Characterization of mrTNF-PADRE

**Figure 6:**
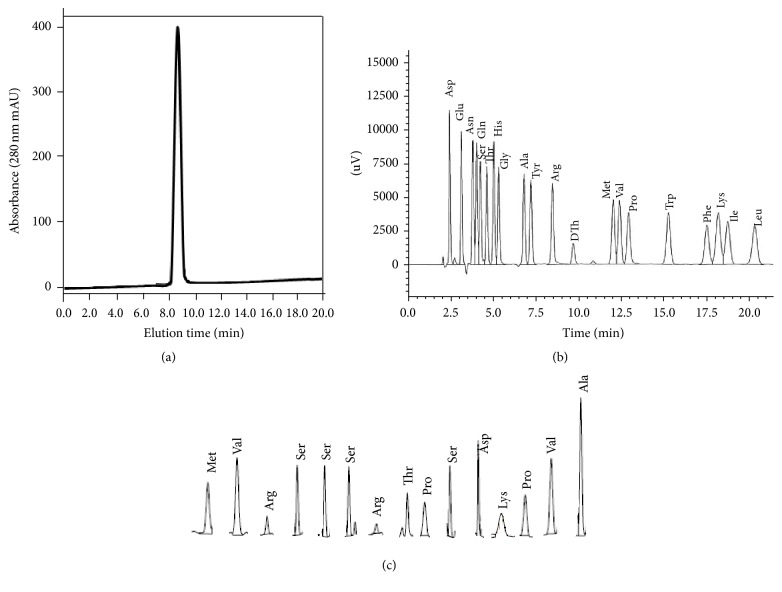
Characterization of mrTNF-PADRE. (a) Analysis of purified mrTNF-PADRE by SEC-HPLC. (b) The amino acid sequencing map of standard samples. (c) The N-terminus 15 amino acids sequencing results of purified mrTNF-PADRE.

The purity of purified mrTNF-PADRE was analyzed by SEC-HPLC, and the purity is over 95% (Figure 6(a)). The N-terminus analysis showed that the sequences of amino acid residues were MVRSSSRTPSDKPVA (Figures 6(b) and 6(c)), consistent with the theoretical sequences.

We also detected the amount of residual* E. coli* proteins and DNAs in each dose of the final purified mrTNF-PADRE vaccine and results showed that the host protein is lower than 0.001% and the DNA is lower than 100 pg (data not shown), which are in accordance with the standard of SFDA.

### 3.5. Polyclonal Antibody Induction in Animals and Neutralization Assay

**Figure 7:**
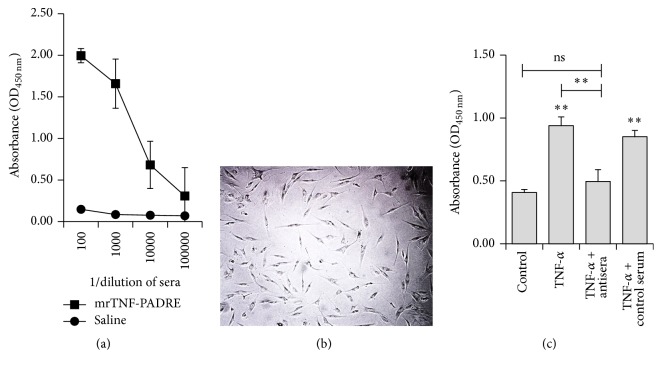
Serum antibody response of mice immunized with mrTNF-PADRE and neutralization assay. (a) TNF-*α*-specific serum antibody responses of mice were measured by ELISA. (b) Primary FLS cells separated from synovial tissues of RA patients. (c) Neutralizing antibody induced by mrTNF-PADRE can inhibit TNF-*α*-stimulated FLS cell proliferation. ^ns^*P* > 0.05 and ^*∗∗*^*P* < 0.01 compared with control group or between indicated groups.

To determine the immunogenicity of mrTNF-PADRE, C57BL/6 mice were immunized at 2-week intervals with the vaccine. Sera were collected and analyzed by ELISA. Result showed that mrTNF-PADRE could induce specific antibodies against human TNF-*α* with the titer reaching a level of 1 : 100,000 (Figure 7(a)).

Neutralization assay showed that the antisera collected from mice significantly inhibited TNF-*α*-stimulated FLS cell proliferation (Figures 7(b) and 7(c)). This indicated that mrTNF-PADRE can induce neutralizing antibodies against TNF-*α*.

## 4. Conclusions

It is well accepted that TNF-*α* plays a critical role in various autoimmune diseases including RA, and various neutralizing TNF-*α* monoclonal antibodies and soluble receptors have been successfully used clinically to treat RA. However, the long-term use of these antagonists could cause some problems like a variable degree of immune tolerance and high costs. In addition, only aiming at a single epitope greatly increased the risk of failure once some mutations were introduced into these epitopes.

One emerging strategy to develop protein vaccines by introducing a T helper cell epitope which acts simultaneously to promote antigen presentation and break the immune tolerance has attracted much attention [14]. By using this strategy, we have previously produced several autovaccines against human proteins in* E. coli* [17–19].

Here, we want to develop an autovaccine to neutralize the endogenous TNF-*α*. To exclude the influence of xenogeneic immunization and immune tolerance on vaccine development, we have previously screened different vaccine construction strategies with mouse TNF-*α* [3, 20]. Finally, a recombinant vaccine mTNF-PADRE was obtained which can adjuvant-freely induce a high titer mouse TNF-*α*-specific neutralizing antibodies which can potently attenuate LPS induced endotoxic shock, TNF-*α* induced cachexia, and collagen-II-induced arthritis (CIA) in mice. In this study, a human TNF-*α* autovaccine was constructed according to this strategy by introducing PADRE to human TNF-*α*. Unfortunately, the recombinant gene rTNF-PADRE failed to be expressed in* E. coli*.

Codon optimization often improves expression of exogenous recombinant genes in* E. coli* via different mechanisms such as preventing the depletion of rare tRNAs and improving the secondary structure and stability of mRNA. We analyzed the gene of rTNF-PADRE and the predicted secondary structure of its mRNA is too complicated to be expressed. Thus, a single codon synonymous mutation (TCT→AGT) was introduced into the gene. As a result, the optimized mrTNF-PADRE vaccine was successfully expressed in* E. coli* at a high level (about 30% of the whole cell lysate).

Taken together, we expressed a human TNF-*α* recombinant vaccine by introducing the epitope PADRE to this protein. The recombinant mrTNF-PADRE was efficiently and stably expressed in* E. coli* at high level and was conveniently purified by a one-step gel filtration method after thorough IBs washing. In addition, the vaccine can induce high titer of neutralizing antibodies which can inhibit TNF-*α*-stimulated FLS proliferation. Considering the fact that TNF-*α* belongs to a huge superfamily and the elicited antibodies may cross-react with other family members, rational designed pharmacodynamics experiments in animal models are still needed.

## Figures and Tables

**Table 1:** Nucleotide sequence of DNA fragments for (m)rTNF-PADRE construction.

Name	Nucleotide sequence
PTF	5′-AATTGGCTAAATTCGTTGCTGCATGGACTCTGAAAGCTGCTCTCGACTTTGCCGAGTCTGGGCAGGTCTACTTTGGGATCATTGCCCTGTGAG-3′

PTR	5′-TCGACTCACAGGGCAATGATCCCAAAGTAGACCTGCCCACTCTCGGCAAAGTCGAGAGCAGCTTTCAGAGTCCATGCAGCAACGAATTTAGCC-3′

P1	5′-GCCATATGGTCAGATCATCTTCTCG-3′

P2	5′-GCGCAATTGGAAGACCCCTC-3′

P3	5′-GCCATATGGTCAGATCATC TTCTCG-3′

P4	5′-GTCGACTCACAGGGCAATGATCCCAAAGTAGACCTGCCC$\underline{\underline{\text{ACT}}}$CTCGGC AAAGTCG-3′

**Table 2:** Typical protein yield of mrTNF-PADRE (100 g of wet cell paste).

Protein expression	TNF-PADRE
Total protein (g)	14.74 ± 0.43
Target protein (g)	4.90 ± 0.27
IBs after washing (g)	1.59 ± 0.27
Solubilized IB (g)	1.40 ± 0.12
Refolded protein (g)	0.52 ± 0.03
Overall yield (%)	10.61
